# Analytic and Monte Carlo calculations of dose-mean lineal energy for 1 MeV–1 GeV protons with application to radiation protection quality factor

**DOI:** 10.1007/s00411-025-01110-w

**Published:** 2025-02-10

**Authors:** Alexis Papadopoulos, Ioanna Kyriakou, Yusuke Matsuya, Miguel Antonio Cortés-Giraldo, Miguel Galocha-Oliva, Ianik Plante, Robert D. Stewart, Ngoc Hoang Tran, Weibo Li, Ioannis A. Daglis, Giovanni Santin, Petteri Nieminen, Sebastien Incerti, Dimitris Emfietzoglou

**Affiliations:** 1 Medical Physics Laboratory, Department of Medicine, University of Ioannina, 45110 Ioannina, Greece; 2 Faculty of Health Sciences, Hokkaido University, Kita-12 Nishi-5, Kita-Ku, Sapporo,, Hokkaido 060-0812 Japan; 3 Nuclear Science and Engineering Center, Research Group for Radiation Transport Analysis, Japan Atomic Energy Agency (JAEA), 2-4 Shirakata, Tokai, Ibaraki 319-1195 Japan; 4 Department of Atomic, Molecular and Nuclear Physics, Universidad de Sevilla, Av Reina Mercedes, s/n. 41012 Seville, Spain; 5 KBR, 2400 NASA Parkway, Houston, TX 77058 USA; 6 Department of Radiation Oncology, University of Washington, Seattle, WA 98195-6043 USA; 7 Radiation Oncology Division, Fred Hutchinson Cancer Center, Seattle, WA USA; 8 UMR 5797, Univ. Bordeaux, CNRS, LP2I, F-33170 Gradignan, France; 9 Federal Office for Radiation Protection (BfS), Ingolstädter Landstraße 1, 85764 Oberschleißheim, Germany; 10 Department of Physics, National and Kapodistrian University of Athens, 15784 Athens, Greece; 11 Hellenic Space Center, 15231 Athens, Greece; 12 ESA/ESTEC Space Environments and Effects Section, ESTEC, Keplerlaan 1, 2200 AG Noordwijk, ZH The Netherlands

**Keywords:** Monte Carlo codes, Quality factor, Analytic models, Microdosimetry, Dose-mean lineal energy, Space radiation

## Abstract

Radiation quality for determining biological effects is commonly linked to the microdosimetric quantity lineal energy ($y$) and to the dose-mean lineal energy (${y}_{\text{D}}$). Calculations of ${y}_{\text{D}}$ are typically performed by specialised Monte Carlo track-structure (MCTS) codes, which can be time-intensive. Thus, microdosimetry-based analytic models are potentially useful for practical calculations. Analytic model calculations of proton ${y}_{\text{D}}$ and radiation protection quality factor ($Q$) values in sub-micron liquid water spheres (diameter 10–1000 nm) over a broad energy range (1 MeV–1 GeV) are compared against MCTS simulations by PHITS, RITRACKS, and Geant4-DNA. Additionally, an improved analytic microdosimetry model is proposed. The original analytic model of Xapsos is refined and model parameters are updated based on Geant4-DNA physics model. Direct proton energy deposition is described by an alternative energy-loss straggling distribution and the contribution of secondary electrons is calculated using the dielectric formulation of the relativistic Born approximation. MCTS simulations of proton ${y}_{\text{D}}$ values using the latest versions of the PHITS, RITRACKS, and Geant4-DNA are reported along with the Monte Carlo Damage Simulation (MCDS) algorithm. The ${y}_{\text{D}}$ datasets are then used within the Theory of Dual Radiation Action (TDRA) to illustrate variations in $Q$ with proton energy. By a careful selection of parameters, overall differences at the ~ 10% level between the proposed analytic model and the MCTS codes can be attained, significantly improving upon existing models. MCDS estimates of ${y}_{\text{D}}$ are generally much lower than estimates from MCTS simulations. The differences of $Q$ among the examined methods are somewhat smaller than those of ${y}_{\text{D}}$. Still, estimates of proton $Q$ values by the present model are in better agreement with MCTS-based estimates than the existing analytic models. An improved microdosimetry-based analytic model is presented for calculating proton ${y}_{\text{D}}$ values over a broad range of proton energies (1 MeV–1 GeV) and target sizes (10–1000 nm) in very good agreement with state-of-the-art MCTS simulations. It is envisioned that the proposed model might be used as an alternative to CPU-intensive MCTS simulations and advance practical microdosimetry and quality factor calculations in medical, accelerator, and space radiation applications.

## Introduction

The analysis and characterization of the microscopic distribution of energy deposition following the interaction of ionizing radiation with the irradiated medium is of significant importance in studying radiation effects for a wide range of applications, including cancer therapy, space radiation protection, as well as single-event errors in electronic devices (United Nations 2010; Durante and Loeffler 2010; Durante et al. 2010; Durante and Cucinotta 2011; Dietze et al. 2013; Cucinotta 2014; Hands et al. 2018). For radiation protection purposes, the radiation quality is commonly linked to the small-scale stochastic pattern of energy deposition which is distinct for each type and energy of radiation (International Commission on Radiation Units and Measurements 1983; Joint Task Group on Radiation Protection Quantities et al. 1986; Protection 2007; Braby et al. 2023). The dependence of biological effects upon radiation quality is formally quantified via the Relative Biological Effectiveness (RBE). For stochastic, mainly carcinogenetic effects, the RBE is synonymous with the quality factor ($Q$)(International Commission on Radiological Protection 1991).

Studies of the physical basis of $Q$ were initially focused on micrometer-sized spherical volumes(Sax 1957; Evans 1962; Goodhead 1982) (diameter ~ 1–10 μm) representative of the cell, cell nucleus and critical chromosomal targets. Currently, there is an increased interest in energy deposition (and/or ionization) events at the nanometer-scale because of the correspondence to the dimensions of the diameter of the deoxyribonucleic acid (DNA) helix, the nucleosome, or sections of chromosomes (Rossi and Zaider 2012; Lindborg and Waker 2020). Energy deposition to such structures is believed to correlate with the RBE for various molecular and cellular endpoints, especially when considering dimensions relevant to the local complexity of DNA damage in the range of 10–20 nm range(Lindborg and Nikjoo 2011; Rossi and Zaider 2012; Lindborg et al. 2013, 2022).

Microdosimetry is commonly employed to study radiation quality issues utilizing the physical quantity lineal energy ($y$) and, specifically, the dose-mean lineal energy (${y}_{\text{D}}$). A biophysical framework for linking ${y}_{\text{D}}$ to RBE (and $Q$) is the Theory of Dual Radiation Action (TDRA)(Kellerer and Rossi 1978; Rossi and Zaider 2012). It is well-known that ${y}_{\text{D}}$ is a better physical indicator of radiation quality to explain biological effects than the conventional, though more practical, Linear Energy Transfer (LET) concept(International Commission on Radiological Protection 1991; Rossi and Zaider 2012; Goodhead 2018; Lindborg and Waker 2020). For example, $y$ is more closely associated with the energy locally deposited to critical molecular and subcellular targets than LET, because the former more accurately accounts for fluctuations of energy-loss and the finite range of secondary electrons. On the other hand, a drawback of the ${y}_{\text{D}}$ concept is that it is challenging to measure or calculate for sub-micron volumes, and such measurements and calculations strongly depend on the target dimensions, geometry and irradiation conditions of interest(Rossi and Zaider 2012).

Monte-Carlo Track-Structure (MCTS) codes, such as GEANT4-DNA(Incerti et al. 2010a, b, 2018; Bernal et al. 2015), KURBUC(Uehara et al. 1993), RITRACKS (Plante andCucinotta 2011) PARTRAC (Friedland et al. 2011), among others(Kyriakou et al. 2022), represent the state-of-the-art in the calculation of ${y}_{\text{D}}$. These codes typically simulate all the main interactions (i.e., ionizations, electronic excitations, and elastic collisions) of particles within the irradiated medium, thus, obtaining the spatial distribution of energy deposition with putative molecular resolution.

Such MCTS simulations incorporate microscopic (or discrete) physical models that may have increased, albeit unknown, levels of uncertainty for low-energy ions and delta-rays, i.e., so-called track-ends. Still, MCTS codes remain today the most accurate tool for obtaining estimates of microdosimetric quantities like ${y}_{\text{D}}$. General purpose Monte-Carlo codes, such as Geant4(Agostinelli et al. 2003; Allison et al. 2006, 2016), PHITS(Sato et al. 2023), MCNP(Rising et al. 2023), FLUKA(Ferrari et al. 2005; Battistoni et al. 2016), and PENELOPE(Salvat et al. 2006), although more rigorously benchmarked than MCTS codes, are not well suited for nanometer (or sub-micron) targets because of limitations of the condensed-history approach. For larger targets (diameter > 1 μm), condensed-history ion-transport methods are potentially useful with the selection of appropriate transport parameters(Stewart et al. 2002).

The importance of microdosimetry in the assessment of radiation quality has recently led several general-purpose MC codes to include track-structure functionality(Uehara et al. 2001; Incerti et al. 2010a, 2018; Fernández-Varea et al. 2012; Goorley et al. 2013; Matsuya et al. 2021, 2022) and hybrid RBE/microdosimetry modelling(Stewart et al. 2015) to be able to switch from a macroscopic (condensed-history) to a microscopic (track-structure) modelling of radiation transport. Published studies suggest that a hybrid approach to practical calculations of ${y}_{\text{D}}$ values is possible by combining small-scale analytic models with larger-scale condensed history models to account for geometry and tissue heterogeneities(Stewart et al. 2015). Hybrid microdosimetry models combine the specifics of the irradiation geometry with the smaller-scale transport physics necessary for the accurate accounting of energy-loss straggling and the finite range of secondary electrons in small volumes. There have been several microdosimetry-based analytic models for charged particles(Wilson et al. 1988; Olko and Booz 1990; Xapsos et al. 1994, 1996; Wilson and Paretzke 1994; Czopyk and Olko 2006; Stewart et al. 2011), each one developed and applied for a specific energy range, target medium and a specific or limited range of ion types.

The objective of the present study is to: *(i)* compare various analytic models for calculating proton ${y}_{\text{D}}$ values against MCTS simulations by three state-of-the-art codes (PHITS, RITRACKS, and Geant4-DNA) as well as the quasi-deterministic microdosimetry algorithm included in MCDS (the scope of the comparisons includes liquid water spheres with diameters in the range 10–1000 nm over the proton energies 1 MeV–1 GeV, which is relevant to radiotherapeutic and space applications); *(ii)* propose an improved analytic model that exhibits better agreement with the MCTS simulation data for ${y}_{\text{D}}$ compared to existing models; and *(iii)* utilize the ${y}_{\text{D}}$ datasets generated by the above methods to investigate the variations of the radiation protection quality factor ($Q$) with proton energy and the method of calculation.

## Material and methods

It is well documented in the literature that equal values of the absorbed dose ($D$) for different types and energies of ionizing radiation do not create the same levels of molecular and cellular damage. These observations are often related to the small-scale distribution of energy deposition events, which motivates the use of LET to determine RBE and $Q$, e.g., as LET increases, biological damage (as well as $Q$ and RBE) at the molecular, cellular, and tissue levels tends to increase.

### Microdosimetric calculation of radiation quality

Deterministic quantities, such as $D$ and LET, do not capture stochastic aspects of the interactions of charged particles with the medium in small (~ nm–μm) volumes. The stochastic fluctuations of energy deposition and the finite range of secondary (delta) electrons in small target volumes of interest for assessments of molecular and cellular damage may be crucial and, in general, need to be considered(Kellerer and Chmelevsky 1975; International Commission on Radiation Units and Measurements 1983; Kellerer 1984b, 1985; Rossi and Zaider 2012).

In microdosimetry, the stochastic analogues of $D$ and LET are represented by the specific energy ($z$) and the lineal energy ($y$), respectively. Lineal energy is the quotient of the energy imparted by a single primary particle and/or its secondary particles (delta electrons and/or secondary ions), inside a target volume divided by the mean chord length ($\overline{l }$) of that volume(Rossi and Zaider 2012):

1 $$y = \frac{{\mathop \sum \nolimits_{i} \varepsilon_{i} }}{{\overline{l}}}$$

where the summation is over all the individual stochastic energy deposits (${\varepsilon }_{i}$). Lineal energy is usually expressed in units of keV/μm, while the mean chord length for a sphere is $\overline{l }=2d/3$, where $d$ is the sphere’s diameter. This mean value of $\overline{l }$ results from the so-called $\mu$-randomness, in which the target is isotropically and uniformly intersected by infinite straight lines(Kellerer 1984a; Rossi and Zaider 2012). To a good approximation, this condition holds for experimental microdosimetry, external beam radiotherapy, and for space radiation.

Concerning radiation quality, it is of interest to define two average values of $y$, namely the frequency-mean $({y}_{\text{F}})$ and the dose-mean $({y}_{\text{D}})$ lineal energy which are described by the following equations(International Commission on Radiation Units and Measurements 1983; Kellerer 1984b; Rossi and Zaider 2012):

2 $$y_{{\text{F}}} = \int yf\left( y \right){\text{d}}y$$

and

3 $$y_{{\text{D}}} = \frac{1}{{y_{{\text{F}}} }}\int y^{2} f\left( y \right){\text{dy}}$$

The quantity ${y}_{\text{D}}$, plays a key role in the Theory of Dual Radiation Action (TDRA) (Rossi and Zaider 2012). In its original and most practical formulation (the so-called site model), it yields the following general expression for the RBE(Rossi and Zaider 2012; Kyriakou et al. 2021):

4 $$\begin{aligned}&{\text{RBE}}_{{{\text{TDRA}}}}\\&\;\; = \frac{{\sqrt {(c \times y_{{{\text{D}},{\text{ref}}}} )^{2} + 4D_{{{\text{test}}}} \left( {c \times y_{{{\text{D}},{\text{test}}}} + D_{{{\text{test}}}} } \right)} - (c \times y_{{{\text{D}},{\text{ref}}}} )}}{{2D_{{{\text{test}}}} }} \end{aligned}$$

where the subscripts “ref” and “test” refer to the reference and test radiations, respectively, and $c$ is a normalization constant(Rossi and Zaider 2012; Kyriakou et al. 2021). In the limit of low doses ($D\to 0$ and assuming ${D}_{\text{test}}/c\ll {y}_{\text{D},\text{ref}}$), the RBE of Eq. (4) may be identified with the quality factor ($Q$), yielding:

5 $$Q_{{{\text{TDRA}}}} = \frac{{y_{{{\text{D}},{\text{test}}}} }}{{y_{{{\text{D}},{\text{ref}}}} }}$$

For studying the variation of $Q$ with the proton energy we have set as reference radiation the proton energy of 100 MeV. This choice is made here for convenience, as some of the ${y}_{\text{D}}$ datasets used have an upper limit of 100 MeV. It is clear from Eq. (1) that lineal energy also depends upon the dimensions of the target under study via $\overline{l }$.

#### New microdosimetry-based analytic model

Following Xapsos and colleagues (Xapsos et al. 1996) the present microdosimetric model considers both direct and indirect events. Direct events (or crossers) occur when a charged particle crosses through the volume of interest(International Commission on Radiation Units and Measurements 1983; Kellerer 1985; Olko and Booz 1990; Xapsos et al. 1994). Charged particles may also pass outside the target and ionize the surrounding material so that delta-ray electrons can reach the target and dissipate their energy inside it; this is termed an indirect event or toucher(International Commission on Radiation Units and Measurements 1983; Xapsos et al. 1994). According to this scheme, the total ${y}_{\text{D}}$ from direct and indirect events can be expressed as(Xapsos et al. 1994):

6 $$y_{{\text{D}}} = f_{{{\text{ion}}}} \times y_{{{\text{D}},{\text{dir}}}} + \left( {1 - f_{{{\text{ion}}}} } \right) \times y_{{{\text{D}},{\text{ind}}}}$$

The above equation indicates that the total ${y}_{\text{D}}$ arises from both direct (${y}_{\text{D},\text{dir}}$) and indirect (${y}_{\text{D},\text{ind}}$) contributions which are combined with an appropriate weighting factor, ${f}_{\text{ion}}$(Xapsos et al. 1994). The calculation of the various terms of Eq. (6) is discussed below.

#### Direct events

The main physical inputs are the average deposited energy by protons in the target, the relative variance of the energy-loss straggling distribution, and the path length and LET fluctuations(Kellerer 1985). In this work, we investigate alternative energy-loss straggling distributions guided from the MCTS simulation data obtained by Geant4-DNA, RITRACKS and PHITS, while the so-called straggling factor (${\delta }_{2}$; the subscript “2” is historically used by Kellerer (Kellerer 1985) to denote the use of the second moment of the collision spectrum) is calculated from first principles within the Relativistic Plane Wave Born Approximation using a dielectric response function for liquid water(Kyriakou et al. 2015). The spatially restricted LET and the weighting factors for direct (and indirect) are determined by the approach of Xapsos(Xapsos 1992; Xapsos et al. 1994). Specifically, to determine the average energy deposited from direct events, it is assumed that a spherical target of 10–1000 nm is randomly crossed by a proton in the energy range of 1 MeV–1 GeV. The proton dissipates energy to the target in a discrete manner, mainly through inelastic Coulomb interactions with the atomic electrons of the medium. The ionized atomic electrons (called secondary electrons) can, in turn, lose their energies inside or outside the target, depending on their kinetic energy. To a first approximation, the average energy deposited by protons inside a target volume can be described by the product of the proton $\text{LET}$ and the mean chord length of that volume ($\overline{l }$)(Kellerer 1985; Rossi and Zaider 2012). However, as the proton energy increases and the target dimension becomes smaller (μm–nm), the number of inelastic collisions with atomic electrons decreases and, in addition, more delta electrons may escape the target. Consequently, a restricted form of LET (L_Δ_) and an energy-loss straggling distribution ($\delta$) must be considered. For the average energy imparted to the target, we can utilize the following equation(Xapsos et al. 1996):

7 $$\overline{\varepsilon } = f_{{{\text{ion}}}} \times {\text{LET}} \times \overline{l}$$

where ${f}_{\text{ion}}$ is the fraction of the proton energy-loss deposited inside the volume. It is calculated by dividing the spatially restricted LET (${\text{L}}_{\Delta }$) with the unrestricted LET of the proton, while adding corrections related to the energy that is carried outside the target by delta electrons. It is here calculated by (Xapsos 1992; Xapsos et al. 1996):

8 $$f_{{{\text{ion}}}} = \frac{{{\text{ln}}\left[ {\frac{{E_{{{\text{max}}}} \left( {\Delta + \Delta_{1} + \Delta_{2} } \right)}}{{I^{2} }} } \right]}}{{2{\text{ln}}\left[ {\frac{{E_{{{\text{max}}}} }}{I}} \right]}}$$

where $I$ is the mean excitation energy of the medium (here taken as $I=0.078 keV$(International Commission on Radiation Units and Measurements 2014), and ${\rm E}_{\text{max}}$ is the maximum energy of a delta electron following a single proton collision(Salvat 2013):

9 $$E_{{{\text{max}}}} = \frac{{2mc^{2} \beta^{2} \gamma^{2} }}{{1 + 2\gamma \left( \frac{m}{M} \right) + \left( \frac{m}{M} \right)^{2} }} \approx 2mc^{2} \beta^{2} \gamma^{2}$$

$\Delta$ is the cut-off energy of electrons with range (${R}_{\text{el}}$) equal to the mean chord length ($\overline{l }$):

10 $$R_{{{\text{el}}}} \left( \Delta \right) = \overline{l}$$

${\Delta }_{1}$ corresponds to the fraction of proton energy- loss retained in the site by delta electrons which are generated inside the volume but mitigate out of it. ${\Delta }_{2}$ accounts for the proton’s energy loss due to ionization binding energy (and excitation transition energy) when the generated delta electrons escape the volume. It may be shown that the following equation hold(Xapsos et al. 1996):

11 $$\Delta_{1} + \Delta_{2} = \left( {1 - \frac{\Delta }{{E_{{{\text{max}}}} }}} \right)\left( {I + \Delta } \right)$$

Regarding the energy loss straggling distribution, which is incorporated into Eq. (6) to calculate the ${y}_{\text{D}}$ contribution of indirect events, there exists several analytical distributions for ions. However, most of them apply only to the limited range of ion energy and target dimensions. Olko and Booz(Olko and Booz 1990) assume a Fermi-like function ($1/({e}^{x}+1))$ for direct events and an exponentially decreasing function for indirect events that was fitted to MC simulation data for proton and alpha particles in the energy range of 0.5–3 MeV and dimensions of 1–1000 nm. A more extensive analytic representation for protons with energy 0.3–20 MeV was made by Wilson(Wilson et al. 1988) and Wilson and Paretzke(Wilson and Paretzke 1994), suggesting a two-parameter Log-normal distribution for direct events and an exponential function for indirect events applicable to spheres with diameter from 2–100 nm. The Log-normal parameters of the mean and the variance were considered as free parameters fitted to their MC simulations. The microdosimetric model of Xapsos(Xapsos et al. 1994, 1996; Shinn et al. 1999; Badavi et al. 2009) has been extensively applied to Tissue Equivalent Proportional Counter (TEPC) measurements for space radiation environments. For this application, a log-normal energy-loss straggling distribution has been utilized and applied to spheres with diameters in the range 2–1000 nm. This distribution had been selected since some early ^60^Co experiments and proton MC data in the energy range of 0.3–20 MeV were well represented by a Log-normal distribution. Recently, an updated Xapsos model with a log-normal energy-loss straggling distribution was presented and applied to protons up to 250 MeV in water liquid spheres of 10–1000 nm(Papadopoulos et al. 2022). The resulting microdosimetric calculations (${y}_{\text{D}})$ showed good agreement with recent MCTS simulation data, although at higher energies (> 50–100 MeV) and nanometer dimensions (10–100 nm) sizeable discrepancies were observed.

Despite the above developments, microdosimetric calculations for particle radiotherapy and radiation in outer space environments require comprehensive proton and ion ${y}_{\text{D}}$ values, over a wide spectrum of energies and target dimensions. For example, proton data for energies at least up to 250 MeV (radiotherapy) or 10 GeV (space radiation protection) are needed(Durante 2004, 2014; Rossi and Zaider 2012; Dietze et al. 2013; Reitz 2018; Chancellor et al. 2021; Cucinotta 2010). Towards extending the applicability of the present model to a wide range of proton energies (from MeV to GeV) and target dimensions (from μm down to nm), we have examined the implementation of various statistical distributions (Log-normal, Erlang, and Logistic) for representing the proton energy-loss straggling.

The probability density function (PDF) of the Log-normal distribution for the energy straggling ($x$), with parameters ${\mu }_{lgn}$ and ${\sigma }_{lgn}$ is given by:

12 $$p\left( {x;\mu_{lgn} ,\sigma_{lgn} } \right)_{lgn} = \frac{1}{{\sqrt {2\pi } \sigma_{lgn} x}}e^{{ - \left( {(lnx - \mu_{lgn} )/\sqrt 2 \sigma_{lgn} } \right)^{2} }}$$

where ${\mu }_{lgn}$ and ${\sigma }_{lgn}$ are the mean and variance of the distribution, respectively. They can be calculated from(Xapsos et al. 1996):

13 $$\mu_{lgn} = \ln \left( {\overline{\varepsilon }} \right) - 0.5\sigma_{lgn}^{2}$$

14 $$\sigma_{lgn} = \sqrt {{\text{ln}}\left( {1 + V} \right)}$$

where $\overline{\varepsilon }$ is the average energy deposited to the site (Eq. (7)) and $V$ is the relative variance of the random processes, namely the energy-loss straggling, the path length and LET fluctuations.

The PDF of the Erlang distribution for the energy-loss straggling ($x$), with shape parameter $\kappa$ and rate parameter $\lambda$, reads:

15 $$p\left( {x;\lambda ,\kappa } \right)_{Erl} = \frac{{\lambda^{\kappa } }}{{\left( {\kappa - 1} \right)!}}x^{\kappa - 1} e^{ - \lambda x}$$

The parameters $\lambda$ and $\kappa$ relate to the mean and the variance of the Erlang distribution which, in turn, are related to the mean energy deposited to the site ($\overline{\varepsilon }$) and the relative variance ($V$) of the random processes. The equations for the parameters $\lambda$ and $\kappa$ can be obtained from:

16 $$\lambda = \frac{1}{{V \times \overline{\varepsilon }}}$$

17 $$\kappa = \frac{1}{V}$$

The PDF of the logistic distribution for the energy-loss straggling ($x$), with mean parameter ${\mu }_{log}$ and scale parameter $s$ is:

18 $$p\left( {x;\mu_{log} ,s} \right)_{Log} = \frac{1}{4s}sech^{2} \left( {\frac{{x - \mu_{log} }}{2s}} \right)$$

In a manner analogous to the Log-normal and Erlang distributions, the parameters ${\mu }_{log}$ and $s$ are associated with the mean energy deposited to the target ($\overline{\varepsilon }$) and the relative variance ($V$) through the following expressions:

19 $$\mu_{log} = \overline{\varepsilon }$$

20 $$s = \frac{\sqrt 3 }{\pi }\overline{\varepsilon } \times \sqrt V$$

The relative variance $V$ needed in Eqs. (14), (16–17) and (20) is calculated by the following equation:

21 $$V = V_{s} + V_{s} \times V_{{{\text{LET}}}} + V_{\delta } + V_{{{\text{LET}}}} \approx V_{\delta } + V_{s}$$

${V}_{\text{LET}}$ is the relative variance of the particle LET as it crosses the volume under study. For the energy range of protons (1 MeV–1 GeV) and the sphere diameters (10–1000 nm) considered in this work, the proton energy loss is small compared to its kinetic energy. Therefore, the LET value for each proton energy does not change significantly across the target volume, so there are no LET fluctuations for monoenergetic proton beams, i.e., ${V}_{\text{LET}}\approx 0$(Xapsos et al. 1994).

${V}_{s}$ represents the fluctuations of the ion path length. Protons in the present energy range (1 MeV–1 GeV) have a much larger range than the target dimensions, so it may be assumed that they cross the target in straight lines(Kellerer and Chmelevsky 1975; Kellerer 1985). As a result, the fluctuations in the proton track length distribution can be determined if their path line equation is known. Assuming a track length distribution for a sphere, $c\left(l\right)=2l/{d}^{2}$, it follows that ${V}_{s}$ is constant and equal to $0.125$(Kellerer 1985).

${V}_{\delta }$ represents the relative variance of the energy-loss straggling of delta electrons. This parameter depends upon the delta influx and efflux since not all secondary electrons would deposit their energy locally and some may escape the target, especially for nanometer dimensions(Kellerer 1985; Xapsos et al. 1994; Rossi and Zaider 2012). In general, for energies much larger than the binding energies, the secondary electron spectrum is assumed to follow a $1/{\rm E}^{2}$ pattern, where E represents the kinetic energy of the electron, once it is set in motion after the ionization. However, this does not hold for very low-energy electrons which have energies comparable to atomic binding energies(Emfietzoglou 2003). So, the difficulty for an accurate consideration of the energy-loss straggling distribution of delta electrons lies in determining the single-collision spectrum, especially for low-energy transfers. ${V}_{\delta }$ is commonly represented by the dose-weighted energy that is deposited in the target in a single collision (${\delta }_{2}$)(Kellerer 1985; Xapsos et al. 1994).

22 $$V_{\delta } = \frac{{\delta_{2} }}{{\overline{\varepsilon }}}$$

where $\overline{\varepsilon }$ is the average energy deposited in the target (Eq. (7)). In the present work, ${\delta }_{2}$ is calculated from first principles according to the expression(Kellerer 1985):

23 $$\delta_{2} = \frac{{\mathop \sum \nolimits_{n}^{ioniz.} \mathop \int \nolimits_{0}^{\Delta } (E + B_{n} )^{2} \frac{{d\sigma_{{{\text{RPWBA}}}}^{\left( n \right)} }}{dE} dE}}{{\mathop \sum \nolimits_{n}^{ioniz.} \mathop \int \nolimits_{0}^{\Delta } \left( {E + B_{n} } \right) \frac{{d\sigma_{{{\text{RPWBA}}}}^{\left( n \right)} }}{dE} dE}}$$

where ${d\sigma }_{\text{RPWBA}}^{(n)}/dE$ is the differential ionization cross section (DICS) in secondary electron energy for the *n*-th ionization shell calculated within the relativistic plane wave Born approximation (RPWBA) (see below), ${B}_{n}$ is the binding energy of the *n*-th ionization shell, and the upper limit of the integrals includes the cut-off energy $\Delta$ to account only for energy losses that remain to the target (Xapsos et al 1996). The $\Delta$ values were obtained for each sphere diameter by Geant4-DNA simulations for electron penetration depths with the condition that $\Delta$ is the electron energy which range equals mean chord length of the target volume (see Eq. (10))(Kyriakou et al. 2016; Papadopoulos et al. 2022). Τhe following values were obtained: Δ = 5.56 keV for d = 1000 nm, Δ = 1.37 keV for d = 100 nm, and Δ = 0.180 keV for d = 10 nm.

The total probability density function for the energy loss ($x$) in the target is the convolution of the energy-loss straggling distribution ($p(x;\lambda ,\kappa )$) of each proton energy and path length $l$, with the chord length distribution, $c(l)$ (Xapsos et al. 1996):

24 $$f\left( {x,l} \right) = \int p\left( x \right)c\left( l \right)dl$$

Then, Eq. (24) with the appropriate energy-loss straggling distribution (Log-normal, Erlang, Logistic), may be used in Eqs. (2) and (3) to calculate the ${y}_{\text{D}}$ for direct events.

#### Indirect events

In the case of indirect events, delta electrons created from ions that pass outside the target, may reach and deposit energy inside the target. For sufficient small targets, there is also a chance that energetic secondary electrons escape the volume. Describing the secondary electron spectrum analytically is a difficult theoretical task. Xapsos and co-workers(Xapsos et al. 1994) suggested an approach that is analogous to that of the direct events by calculating the average energy deposited by electrons in the target and the fluctuations of the three random factors ($\text{LET}, l, \delta )$. In that approach, the mean electron LET is determined by assuming a $1/{E}^{2}$ initial electron energy spectrum (produced by the ion) and $1/{\text{LET}}_{e}(E)$ average LET slowing down electron spectrum.

In the present work for each proton energy, the mean ${y}_{\text{D}}$ of indirect events is calculated as follows: when a monoenergetic proton ionizes the medium (outside the target), there is a probability $\{{P}_{1},{P}_{2},\dots \}$ of ejecting electrons with energies $\{{E}_{1},{E}_{2},\dots \}$. This probability is determined by the DICS (see next section). Then, each electron with energy ${E}_{i}$, will ionize other electrons of the target, resulting in a value of ${y}_{\text{D},i}$. According to this approach, secondary electrons can be characterized by the triplet $\{{P}_{i},{E}_{i},{y}_{\text{D},i}\}$ associated with their probability of ejection (${P}_{i}$), kinetic energy (${E}_{i}$) and the corresponding ${y}_{\text{D},i}$ values, respectively. In the present work, the ${y}_{\text{D}}$ values for each electron energy and sphere diameter were pre-calculated via Geant4-DNA simulations for monoenergetic electrons with initial energy covering the whole secondary electron spectrum. Then, the mean ${y}_{\text{D},\text{ind}}$ for indirect events for each proton energy (1 MeV–1 GeV) and target dimension (10 nm, 100 nm, and 1000 nm), can be calculated from the following integral:

25 $$y_{{{\text{D}},{\text{ind}}}} = \frac{{\mathop \sum \nolimits_{n}^{ioniz.} \mathop \int \nolimits_{\Delta }^{{E_{{{\text{max}},n}} }} y_{{\text{D}}} \left( {E,d} \right)\frac{{d\sigma_{{{\text{RPWBA}}}}^{\left( n \right)} }}{{{\text{d}}E}}{\text{ d}}E}}{{\mathop \sum \nolimits_{n}^{ioniz.} \mathop \int \nolimits_{\Delta }^{{E_{{{\text{max}},n}} }} \frac{{d\sigma_{{{\text{RPWBA}}}}^{\left( n \right)} }}{{{\text{d}}E}}{\text{ d}}E}}$$

where ${E}_{\text{max},n}=\frac{E+{B}_{n}}{2}$ and ${d\sigma }_{\text{RPWBA}}^{(n)}/dE$ is the DICS in secondary electron energy for the *n*-th ionization shell (see below). The denominator in Eq. (25) is used for normalization purposes. The lower integration limit $\Delta$ of the above equation is the appropriate geometrical cut-off energy for electrons, based on the Spencer-Attix considerations(Spencer and Attix 1955; Xapsos et al. 1994). Note that the same *Δ* values employed in Eq. (23) are utilized in Eq. (25) for the sphere diameters of 10, 100, and 1000 nm.

### Proton inelastic cross sections

To address the interaction between energetic protons and atomic electrons in liquid water, the dielectric formulation of the Relativistic Plane Wave Born Approximation (RPWBA) is considered the state-of-the-art in the field(Salvat 2013). This approach is used to calculate the DICS which is needed to calculate important physical parameters of the model as described above (e.g., proton LET, ${\delta }_{2}$, and ${y}_{\text{D},\text{ind}}$). As mentioned above, the main energy-loss mechanism of protons in the energy domain considered here is the inelastic interaction with the atomic electrons. For condensed phase (liquid or solid) water, the bare Coulomb interactions between protons and atomic electrons are affected by the long-range polarization and screening effects of the medium. These effects are known to (strongly) influence the electronic excitation spectrum, especially the low energy-loss part of the spectrum. The above effects are commonly considered via the complex dielectric response function (DRF), $\varepsilon (W,q)={\varepsilon }_{1}(W,q)+i{\varepsilon }_{2}(W,q)$ of the medium with $W$ and $q$ being the energy transfer and recoil energy (or momentum transfer), respectively. Note that $W$ equals $W\equiv E+{B}_{n}$ in the case of ionizations. In the context of the RPWBA, the only non-trivial part in the calculation of DICS is the energy-loss -function (ELF) defined as(Ritchie 1982):

26 $${\text{ELF}} \equiv {\text{Im}}\left[ { - \frac{1}{{\varepsilon \left( {W,q} \right)}}} \right] = \frac{{\varepsilon_{2} \left( {W,q} \right)}}{{\left| {\varepsilon \left( {W,q} \right)} \right|^{2} }}$$

Many different approaches have been presented in the literature for calculating the ELF of liquid water(Emfietzoglou et al. 2005, 2017; Dingfelder 2014; Kyriakou et al. 2015; Garcia‐Molina et al. 2017). In the present work we adopt the latest model implemented in Geant4-DNA for electrons (Kyriakou et al. 2022) and apply it to protons. In this approach, the real part of the DRF is decomposed to the individual ionization shells ($n$) and excitation levels ($k$) of liquid water yielding the following expression for the ELF:

27 $${\text{ELF}} = {\text{ELF}}_{{{\text{ioniz}}}} + {\text{ELF}}_{{{\text{excit}}}} = \mathop \sum \limits_{n}^{ioniz.} \frac{{\varepsilon_{2}^{\left( n \right)} \left( {W,q} \right)}}{{\left| {\varepsilon \left( {W,q} \right)} \right|^{2} }} + \mathop \sum \limits_{k}^{excit.} \frac{{\varepsilon_{2}^{\left( k \right)} \left( {W,q} \right)}}{{\left| {\varepsilon \left( {W,q} \right)} \right|^{2} }}$$

To obtain the dependence of the ELF upon both $W$ and $q$, a Drude-type parameterization of experimental optical data ($q=0$) is extended to non-zero momentum transfers $\left(q>0\right)$ by analytic dispersion relations. The details of the parametrization algorithm and dispersion relations is given elsewhere(Kyriakou et al. 2022). Although the above dielectric model is already implemented into the Geant4-DNA for electron transport(Kyriakou et al. 2022), it is the first time that it is applied to proton inelastic collisions up to relativistic energies.

In RPWBA, the DICS is the sum of two terms(Salvat 2013):

28 $$\frac{{{\text{d}}\sigma }}{{{\text{d}}E}} = \frac{{{\text{d}}\sigma_{{\text{L}}} }}{{{\text{d}}E}} + \frac{{{\text{d}}\sigma_{{\text{T}}} }}{{{\text{d}}E}}$$

where the subscript “L” and “T” refer to the longitudinal and transverse interaction terms, respectively, given by the following expressions(Kyriakou et al. 2022):

29 $$\frac{{{\text{d}}\sigma_{L} }}{{{\text{d}}E}} = \frac{2}{{\pi \alpha_{0} N m c^{2} \beta^{2} }}\mathop \sum \limits_{n}^{ioniz.} \mathop \int \limits_{{q_{{{\text{min}},n}} }}^{{q_{{{\text{max}},n}} }} \frac{{\varepsilon_{2}^{\left( n \right)} \left( {W,q} \right)}}{{\left| {\varepsilon \left( {W,q} \right)} \right|^{2} }}\frac{{\left( {q + mc^{2} } \right)}}{{q\left( {q + 2mc^{2} } \right)}}{\text{d}}q$$

and

30 $$\frac{{{\text{d}}\sigma_{T} }}{{{\text{d}}E}} = \frac{1}{{\pi \alpha_{0} N m c^{2} \beta^{2} }}\mathop \sum \limits_{n}^{ioniz.} \frac{{\varepsilon_{2}^{\left( n \right)} \left( {W,0} \right)}}{{\left| {\varepsilon \left( {W,0} \right)} \right|^{2} }}\left\{ {\ln \left( {\frac{1}{{1 - \beta^{2} }}} \right) - \beta^{2} } \right\}$$

where $${\alpha }_{0}$$ is the Bohr radius, $$N$$ is the density of water molecules in unit density of water, $$\beta$$ is the scaled proton velocity, $$\beta = {\raise0.7ex\hbox{$u$} \!\mathord{\left/ {\vphantom {u c}}\right.\kern-0pt} \!\lower0.7ex\hbox{$c$}}$$, and with $$u$$ as the incident proton velocity. Note that, for the transverse term, we adopt the small-angle scattering approximation and consider only collisions with (nearly) zero-momentum transfer(Fernández‐Varea et al. 2005). The limiting values of the recoil energy are(Salvat 2013):

31 $$\begin{aligned}& q_{{{\text{max}},n/{\text{min}},n}}\\& = \sqrt {\left[ {\sqrt {T\left( {T + 2Mc^{2} } \right)} \pm \sqrt {\left( {T - E - B_{n} } \right)\left( {T - E - B_{n} + 2Mc^{2} } \right)} } \right]^{2} + \left( {mc^{2} } \right)^{2} }\\&\quad - m c^{2}\end{aligned}$$

where $M, T$ is the proton mass and kinetic energy, respectively. The stopping power ($\text{SP}$) (or unrestricted LET) of protons can be calculated within the RPWBA using the expression(2014):

32 $${\text{SP}} = \mathop \sum \limits_{n}^{ioniz.} \mathop \int \limits_{0}^{{E_{{{\text{max}}}}^{\left( n \right)} }} (E + B_{n} )\frac{{d\sigma_{{{\text{RPWBA}}}}^{\left( n \right)} }}{dE}dE + \mathop \sum \limits_{k}^{excit.} B_{k} \sigma_{{{\text{RPWBA}}}}^{\left( k \right)}$$

Note that ${\sigma }_{\text{RPWBA}}^{(k)}$ is simply the integral of Eqs. (28) and (29), whereby the summation over the ionization shells is replaced by a summation over the discrete electronic excitations (*k*) with transition energy ${B}_{k}$.

## Monte Carlo calculation of ${\mathbf{y}}_{\mathbf{D}}$

### Geant4-DNA

The Geant4 toolkit(Agostinelli et al. 2003; Allison et al. 2006, 2016) is a general-purpose Monte Carlo code for the simulation of the passage of particles through matter. It was initially released in 1998 for simulating high energy physics experiments; however, its flexibility and the work of the Geant4 collaboration, allow to be used in many fields like space physics and medical physics. Within Geant4, Geant4-DNA was developed as an extension to model biological damage induced by ionizing radiation at cellular and subcellular scale(Incerti et al. 2010a, b, 2018; Bernal et al. 2015). With this purpose, Geant4-DNA incorporates discrete interaction models of ionizing radiation with liquid water molecules to carry out MCTS simulations.

For this work, the microdosimetric quantities were calculated using the code described in Barrato-Roldan et al.(Baratto-Roldán et al. 2021), compiled with version 11.1.2 (June 2023). A pencil beam of protons was produced at the central point of a side of a box-shaped volume made of water (“world volume”), pointing towards the opposing side. The lateral dimensions of the box were large enough to ensure that all secondary electrons were stopped within the simulated volume. For each proton energy considered, the maximum energy transferred to a secondary electron was calculated and the maximum range ${R}_{\delta ,{\text{max}}}$ was estimated(Tabata et al. 1972). Then, the lateral dimensions of the box, measured from the center, were set slightly larger than ${R}_{\delta ,{\text{max}}}+{d}_{\text{site}}$, with ${d}_{\text{site}}$ being the site diameter, to ensure that the furthest possible random placement of the site lied within the volume(Baratto-Roldán et al. 2021). For example, the transversal dimensions were 2.8 mm for 300 MeV protons and 1.3 µm for 1 MeV protons.

Sites were placed on the proton track following the “weighted” random sampling approach(Bertolet et al. 2019; Baratto-Roldán et al. 2021), which consists in selecting an energy transfer point, placing the spherical site randomly around it and finally scoring the energy imparted by adding all the energy deposits encountered within it. To ensure any secondary electron which would potentially irradiate the site (i.e., those produced a distance smaller than ${R}_{\delta ,{\text{max}}}$ upstream or downstream), we considered for the random placement of the site only the energy transfer points located within a central slab region oriented normally with respect to the proton track. The slab transversal dimensions were equal to those of the world volume. Longitudinally, the slab thickness varied from 0.1 µm (protons at 1 MeV) to 1.0 µm (protons at 300 MeV) to ensure a large enough number of energy transfer points within it and a sufficiently small proton energy loss; further, each boundary of the slab was at a distance ${R}_{\delta ,{\text{max}}}$ from the nearest boundary of the world volume. Therefore, the total size of the world volume along the proton initial direction was twice ${R}_{\delta ,{\text{max}}}+{d}_{site}$, plus the slab thickness. To minimize the impact of the small variation of the proton energy along the slab, the initial energy of the protons was such that the mean value between the proton energy at the entrance and exit of the slab equalled the energy under study.

The physics list used in these simulations was “G4EmDNAPhysics_option2”, the accelerated default physics constructor. This option allows the simulation of proton interactions up to 300 MeV(Domínguez-Muñoz et al. 2022) and electron interactions from 7.4 eV up to 1 MeV(Kyriakou et al. 2022). As for proton transport, the approach considered, depends on its kinetic energy: Drude theory (up to 500 keV), non-relativistic Born approximation (0.5–100 MeV), and RPWBA (100–300 MeV)(Incerti et al. 2010a, b; Domínguez-Muñoz et al. 2022). As for electron interactions, Champion model was used for elastic scattering, and Born model was used for electronic excitation and ionization(Incerti et al. 2010b).

### PHITS-KURBUC

The PHITS track-structure model for protons based on the algorithms of KURBUC code, so-called the PHITS-KURBUC model(Matsuya et al. 2021), was used to calculate the ${y}_{\text{F}}$ and ${y}_{\text{D}}$ values. The PHITS-KURBUC model considers elastic scattering, ionizations (1b_1_, 3a_1_, 1b_2_, 2a_1_, and 1a_1_), excitations (A^1^B_1_, B^1^A_1_, Ryd A + B, Ryd C + D, diffuse band, and collective), dissociative electron attachments (OH($-$), O ($-$), and H($-$) productions), molecular excitations (vibrational (bending and stretching), phonon (vibrational and translational), and rotational excitations (normal and fast components), electron loss, and electron capture. In this calculation, we used the PHITS version 3.31 and set the cut-off energies of protons and electrons as 1 keV and 1 eV, respectively.

When calculating the $y$ distribution, a 10, 100, or 1000 nm diameter water target (i.e., H_2_O at a density of 1.0 g/cm^3^) was placed at the origin surrounded by a 1 cm diameter water which can sufficiently provide secondary electron equilibrium. Because of the available energy range of the PHITS-KURBUC model, the protons with 1 $-$ 300 MeV were incident on the target. In this simulation, we also calculated the indirect events (the energy deposited in the target by secondary electrons) by considering the protons passing outside the target as they slow down. Note that considering the radial dose distribution(Matsuya et al. 2021), we set the radius of the circle plane proton source as 2000 nm. The energy deposition in the water $\varepsilon$ was scored by using the t-deposit tally that allows us to obtain deposition energies in certain regions. After that, the probability densities of lineal energy were obtained using Eq. (1), and the ${y}_{\text{F}}$ and ${y}_{\text{D}}$ Eqs. (2) and (3). The ${y}_{\text{F}}$ values were calculated by using and ${y}_{\text{D}}$ values were calculated with sufficient numbers of particles to make the statistical uncertainty less than 5%.

### PHITS t-sed

The PHITS code also enables the calculation of the microscopic probability densities of lineal energy and specific energy using an analytical function (microdosimetric function) based on the track-structure simulations, named t-sed tally(Sato et al. 2006, 2009). In 2006, the t-sed tally was first developed by using a Monte Carlo track-structure code of TRACEL(Tomita et al. 1997). Recently, PHITS-original track-structure models, i.e., PHITS electron track-structure (PHITS-ETS) and PHITS ion track-structure model for arbitrary materials (ITSART) were developed. From such a development background, the microdosimetric function in the t-sed tally was updated based on the PHITS-ETS and ITSART models(Sato et al. 2023). When simulating the protons with 1–1000 MeV, proton beams were incident to the thin water target (H_2_O, 1.0 g/cm^3^). Note that the target was defined as the thickness at which less than 1% of the energy is deposited. Using the microdosimetric function, we scored the probability densities of lineal energy to calculate the *y*_F_ and *y*_D_ values of a 10, 100, or 1000 nm diameter water sphere. Note that the nuclear reaction models were inactivated in this PHITS calculation. After calculating the lineal-energy distribution, we calculated the ${y}_{\text{F}}$ and ${y}_{\text{D}}$ values with sufficient numbers of particles to make the statistical uncertainty less than 1%.

#### RITRACKS

The code RITRACKS (Relativistic Ion Tracks) simulates the detailed track structure of ions, electrons, and photons in liquid water. The transport algorithms and cross sections used are described in Plante and Cucinotta 2011(Plante and Cucinotta 2011) and references therein. RITRACKS has been used for the calculation of dose in targets(Plante et al. 2011, 2021), radiation chemistry(Plante and Devroye 2017), and chromosome damage(Plante et al. 2019; Slaba et al. 2020). In RITRACKS, an irradiated volume is defined, and targets are placed within it. For this work, the spherical targets were placed at the center of an irradiated volume of the shape of a cylinder, with a radius equal to the radius of the sphere (and length equal to 5 microns). The cylinder was irradiated uniformly by ions impacting the bottom surface. Proton energy is kept constant as they are transported across the cylinder. Periodic boundary conditions were not implemented on the volume, so delta electrons leaving the irradiated volume were not contributing to the dose, except possibly for the rare cases where an electron trajectory could go back and impact the target(Plante et al. 2021). On the other hand, secondary electrons generated inside the irradiated volume, but outside the target, are still allowed to deposit energy to the target. The number of simulation histories varied from 1,200 to 60,000 depending on the size of the target and proton energy, to minimize uncertainty on the results. One track and one target were simulated per Monte-Carlo history. For each simulation history, the energy deposited in the target was recorded. The corresponding lineal energy ($$y)$$ for each target was calculated using Eq. (1). Using the data for all targets, histograms of the quantities $$y$$ and $${y}^{2}$$ were obtained. The average lineal energy $${y}_{\text{F}}$$ was obtained as the mean of the histogram of $$y$$. For $${y}_{\text{D}}$$, the mean of the histogram of $${y}^{2}$$ was used and divided by $${y}_{\text{F}}$$, as in Eq. (3).

#### MCDS

The MCDS was originally developed to estimate the clustering of DNA lesions to form SSB, DSB and other complex types of DNA damage(Semenenko and Stewart 2004, 2006). In 2011, the MCDS was extended to predict DNA damage for a wider range of ions, ion energies and oxygen conditions, i.e., from a pO_2_ value in the range of 0–100%(Stewart et al. 2011). Of note, the MCDS does not rely on any information related to stopping powers, LET, target size or estimates of microdosimetric quantities to estimate the yield of clusters of DNA damage(Semenenko and Stewart 2004, 2006; Stewart et al. 2011, 2015).

The 2011 version of the MCDS (version 3.10A) introduced a deterministic algorithm to estimate microdosimetric quantities (specific energy, lineal energy), CSDA range and related information for ions passing through a pure water $-$ or water equivalent $-$ medium. The MCDS accounts for ion stopping power changes in water for (*Z*_eff_/b)^2^ in the range from 1 to ~ 10^4^–10^5^, corresponding to ^1^H^+^ kinetic energies from at least ~ 1 keV to 1 GeV. Microdosimetric quantities can be calculated for a spherical target surrounded by a vacuum or for a monodirectional beam of ions emitted from a plane that passes through a water-equivalent material before reaching a spherical target. The latter geometry is intended to simulate monolayer cells attached to the bottom of a cell culture dish. The reported MCDS results in this paper are based on the former irradiation geometry.

In the MCDS, ions travel in a straight line until they have zero kinetic energy. The deterministic algorithm used in the MCDS accounts for stoppers within the treatment volume and for changes in ion stopping power within the target volume. It does not account for delta-ray escape from the target (all energy deposition events are considered local) nor does it account for delta-rays associated with ions passing near the target site that enter the site and deposit energy. For irradiation geometries other than the ones described above, the microdosimetry model included in the MCDS should be combined with a larger-scale, Monte Carlo transport simulations to account for secondary radiations(Stewart et al. 2015). Such a hybrid Monte Carlo approach is especially important for indirectly ionizing photons and neutrons.

## Results

Since the RPWBA calculations provide important physical inputs to the present microdosimetric model, we first examine the reliability of these calculations by comparing the present calculations for the proton stopping power and the energy-loss straggling factor (${\delta }_{2}$) against other studies(Kellerer 1985; Xapsos et al. 1994, 2014).

**Fig. 1:**
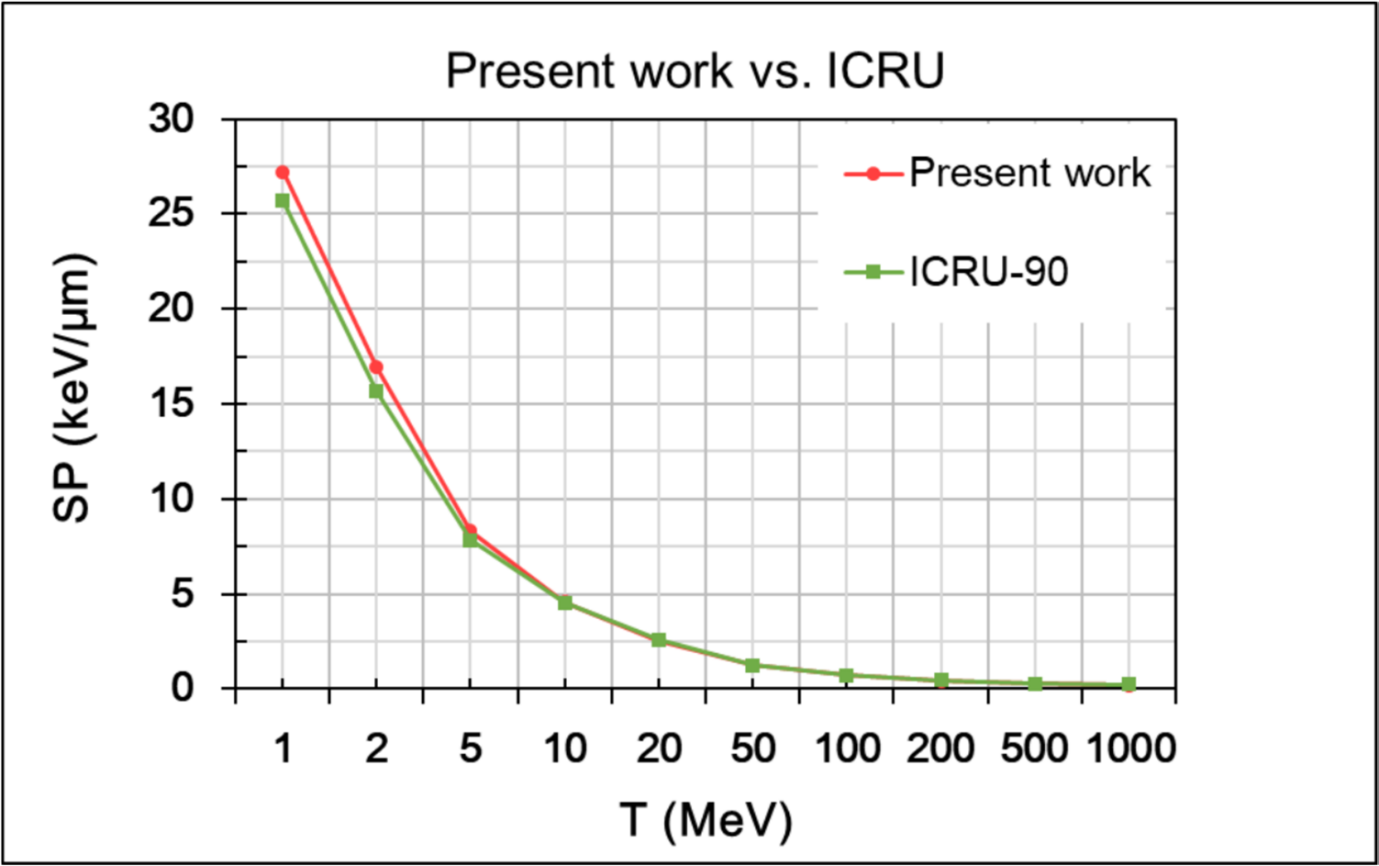
Proton stopping power (SP) for liquid water for the energy range 1 MeV–1 GeV calculated in the present work by the RPWBA and the dielectric theory (red line) and compared against the data from ICRU Report 90 (green line)

Figure 1 shows the proton stopping power (or unrestricted LET) of liquid water calculated from Eq. (32) using the present DRF model combined with the RPWBA for the energy range of 1 MeV $-$ 1 GeV. The calculations are compared against the SP data of the ICRU Report 90( International Commission on Radiation Units and Measurements 2014).

**Fig. 2:**
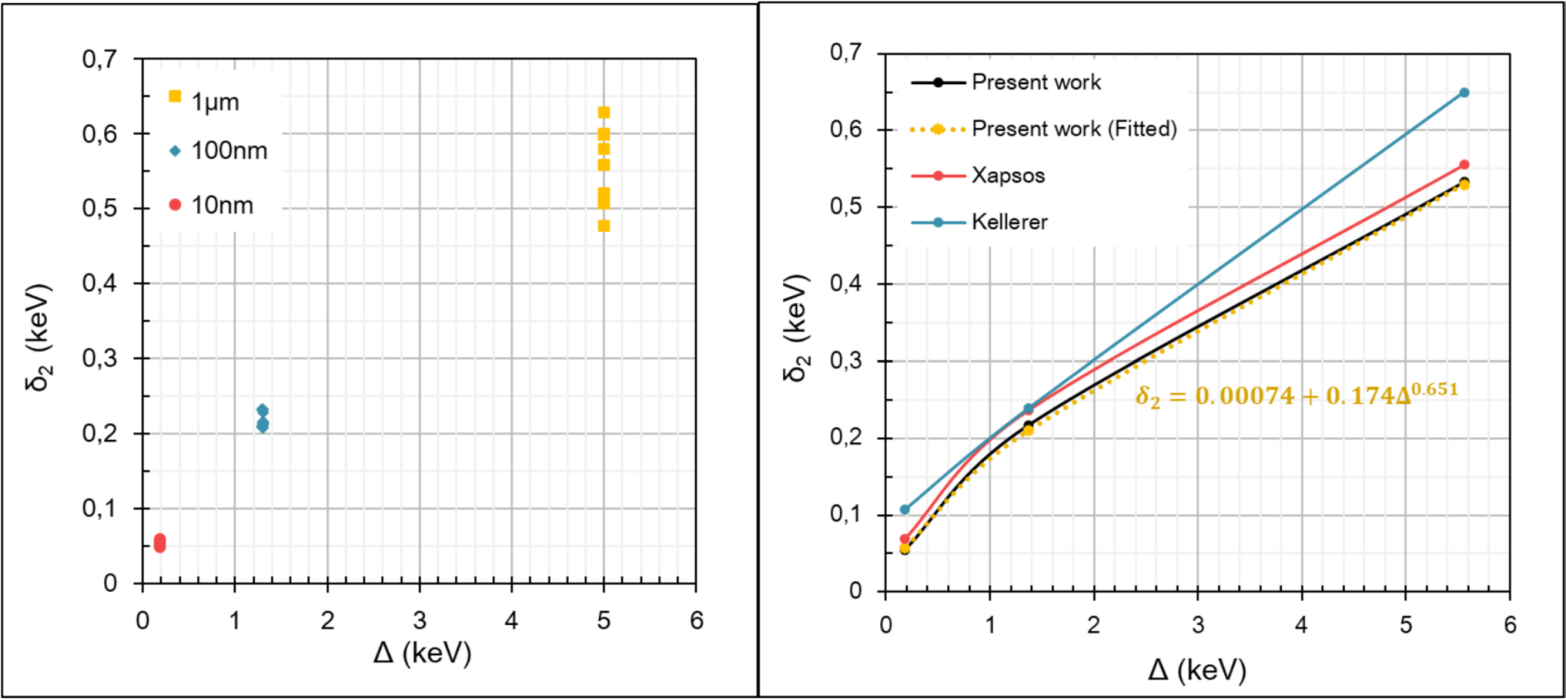
Left panel: Present work ${\delta }_{2}$ values for various proton energies (2, 5, 10, 20, 50, 100, 400, 600, 800, 1000 MeV) and distinct cut-off energies *Δ* corresponding to the three-sphere diameters (10, 100, 1000 nm) obtained from Eq. (23). Right Panel: ${\delta }_{2}$ values as a function of cut off energy $\Delta$, calculated by the approaches of Kellerer(Kellerer 1984b), Xapsos(Xapsos et al. 1994; Papadopoulos et al. 2022) and present work

Figure 2 (left panel) shows the dose-weighted energy that is deposited in the target in a single proton-electron collision (${\delta }_{2}$) calculated by Eq. (23) for various proton energies (2, 5, 10, 20, 50, 100, 400, 600, 800, and 1000 MeV) in the domain of 1 MeV–1 GeV and three target dimensions (10, 100, and 1000 nm) which correspond to distinct cut-off energies $\Delta (0.18, 1.37, \text{and} 5.56 \text{keV})$. The comparison of the present ${\delta }_{2}$ values against the Kellerer (Kellerer 1984b) and Xapsos (Xapsos et al. 1994) approaches is also presented in Fig. 2 (right panel). Our results are represented as a function of the cut-off energy $\Delta$ by the fitted equation ${\delta }_{2}=0.0074+0.174{\Delta }^{0.651} (\text{keV})$ which is valid for $\Delta \le {E}_{max}$. According to a previous study (Papadopoulos et al. 2022) the Xapsos results are reproduced by ${\delta }_{2}=0.195{\Delta }^{0.610}$. Kellerer’s approach assumes a $1/{E}^{2}$ secondary electron spectrum yielding ${\delta }_{2}=\frac{\Delta }{2\text{ln}( \Delta /I)}$, with $I=0.078 \text{keV }$( International Commission on Radiation Units and Measurements 2014).

**Fig. 3:**
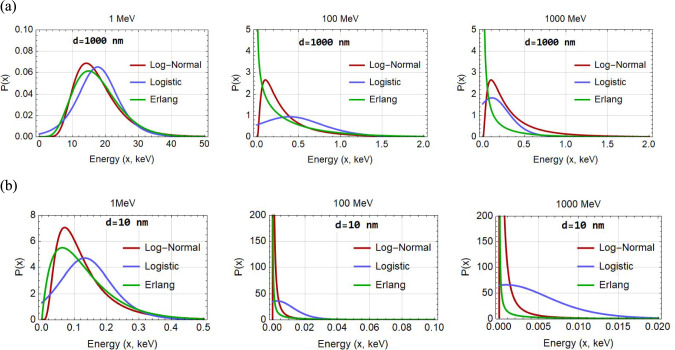
Energy-loss straggling distributions (Log-Normal, Erlang, Logistic) for 1, 100 and 1000 MeV proton energies and sphere diameters of 1000 nm (panel a) and 10 nm (panel b)

Figure 3 depicts the different energy-loss straggling distributions (Log-normal, Erlang, Logistic) for protons (of 1, 100, and 1000 MeV) crossing a liquid water target sphere (diameter equal to 10 and 1000 nm), as implemented into our microdosimetric model for determining ${y}_{\text{D},\text{dir}}$ (i.e., the contribution of direct events in ${y}_{\text{D}}$).

**Fig. 4:**
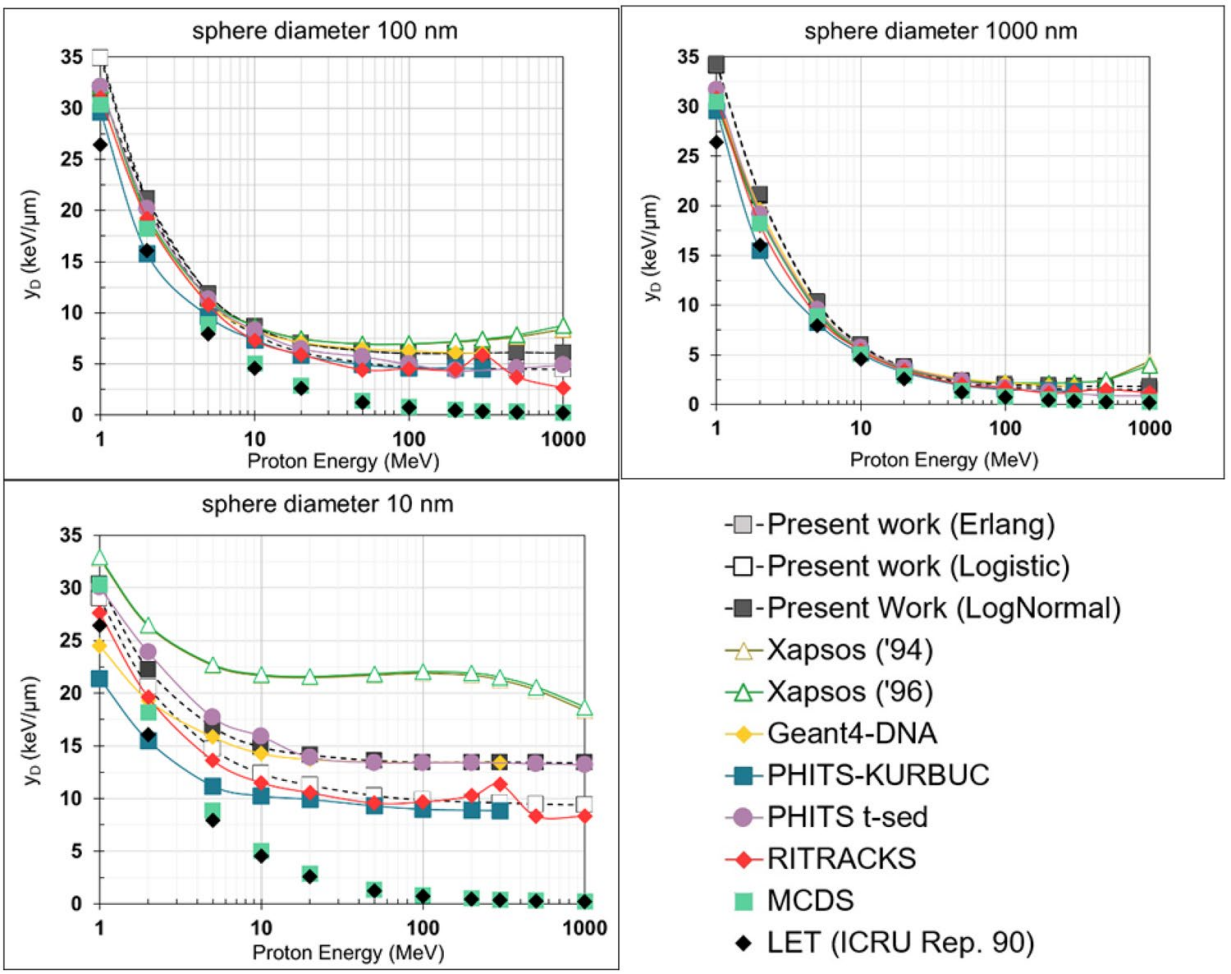
Dose-mean lineal energy (${y}_{\text{D}}$) values for liquid water and various sphere diameters (10, 100, and 1000 nm) as a function of proton energy (1 MeV–1 GeV), calculated by the microdosimetric models (“Present work” and Xapsos models), the MCTS codes (Geant4-DNA, PHITS-KURBUC, PHITS t-sed, and RITRACKS), and the MCDS. The LET values calculated from the ICRU Report 90 are also presented

Figure 4 depicts the ${y}_{\text{D}}$ for the proton energy range 1 MeV–1 GeV and three liquid water spheres of different sizes (10, 100, and 1000 nm). The present calculations and the analytic models of Xapsos and co-workers are compared against MCTS simulations using PHITS (PHITS-KURBUC and PHITS t-sed), Geant4-DNA, and RITRACKS. Data from the deterministic model included in the MCDS is also shown in Fig. 4. Note that the calculations of the ${y}_{\text{D}}$ by the present model are carried out using three different energy-loss straggling distributions (Log-normal, Erlang, and Logistic). Also, the MCDS-based ${y}_{\text{F}}$ values were converted to an estimate of ${y}_{\text{D}}$ using the approximate formula ${y}_{\text{D}}=(9/8){y}_{\text{F}}$, which holds for the idealized case where particles of constant LET traverse the target sphere by losing energy in a continuous manner (International Commission on Radiation Units and Measurements 1983).

**Fig. 5:**
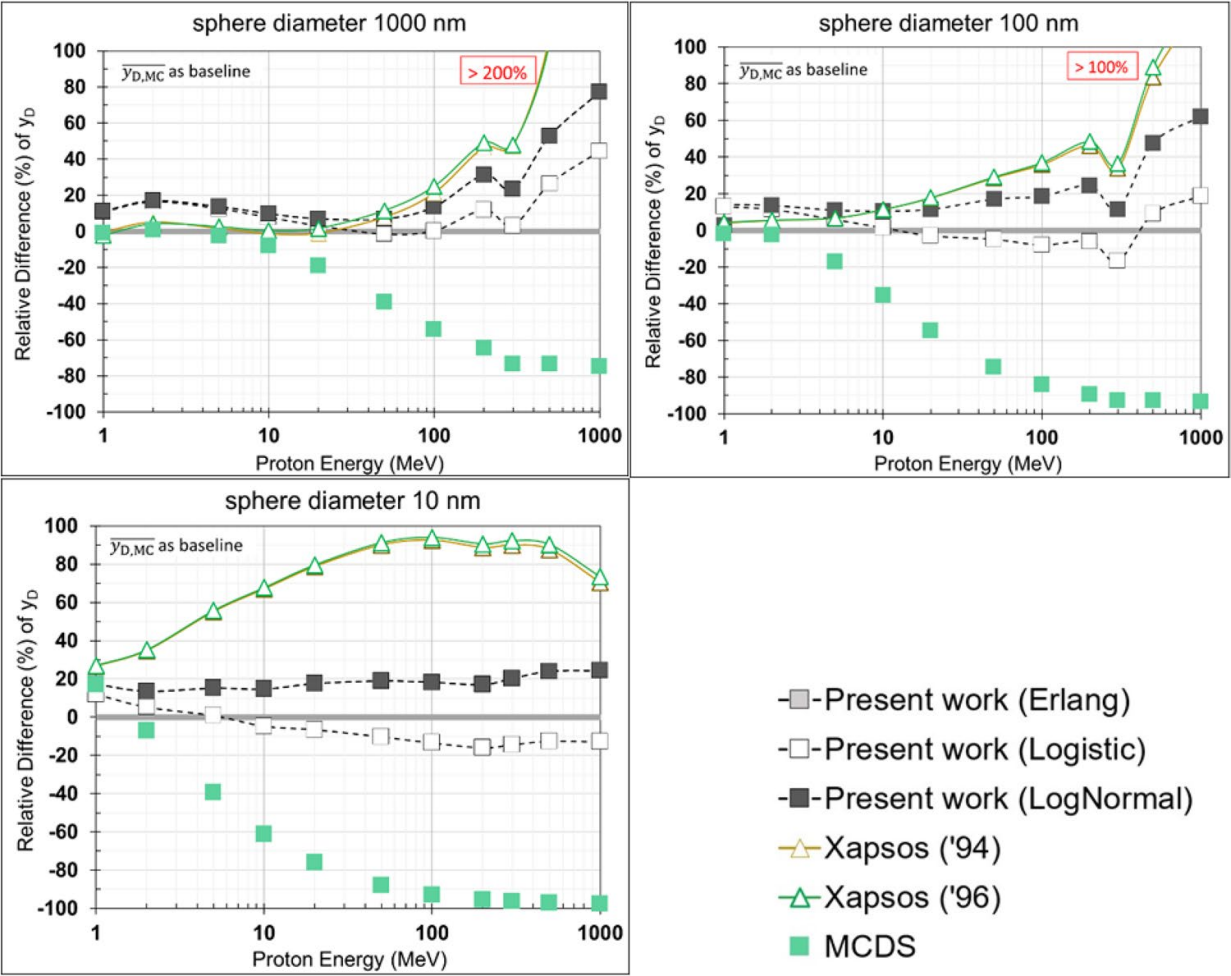
Relative difference (%) of the ${y}_{\text{D}}$ values of the analytic models and the MCDS algorithm against the (arithmetic) mean of the ${y}_{\text{D}}$ values of the MCTS codes for each proton energy (see Fig. 4 and Eq. (33))

Figure 5 depicts for each proton energy (*T*_i_) the relative difference (RD) of the ${y}_{\text{D}}$ values of the analytic models and the MCDS algorithm against the arithmetic mean of the ${y}_{\text{D}}$ values of the MCTS simulations:

33 $${\text{RD}}\left( {T_{{\text{i}}} } \right) = \frac{{y_{{\text{D}}} \left( {T_{{\text{i}}} } \right) - \overline{{y_{{{\text{D}},{\text{MCTS}}}} \left( {T_{{\text{i}}} } \right)}} }}{{\overline{{y_{{{\text{D}},{\text{MCTS}}}} \left( {T_{{\text{i}}} } \right)}} }} \times 100\%$$

where *T*_i_ is the i-th discrete energy value in the 1 MeV–1 GeV interval and $\overline{{y }_{\text{D},\text{MCTS}}\left({T}_{\text{i}}\right)}$ is the (arithmetic) mean of the ${y}_{\text{D}}$ values of the MCTS codes at energy *T*_i_. Note that the upper limit of application of the Geant4-DNA and PHITS-KURBUC is 300 MeV, while the upper limit of PHITS t-sed and RITRACKS is 1 GeV. Therefore, the value of $\overline{{y }_{\text{D},\text{MCTS}}\left({T}_{\text{i}}\right)}$ for *T*_i_ < 300 MeV is based on Geant4-DNA, PHITS-KURBUC, PHITS t-sed, and RITRACKS, whereas the value of $\overline{{y }_{\text{D},\text{MCTS}}\left({T}_{\text{i}}\right)}$ for *T*_i_ > 300 MeV is based only on data by RITRACKS and PHITS t-sed.

**Fig. 6:**
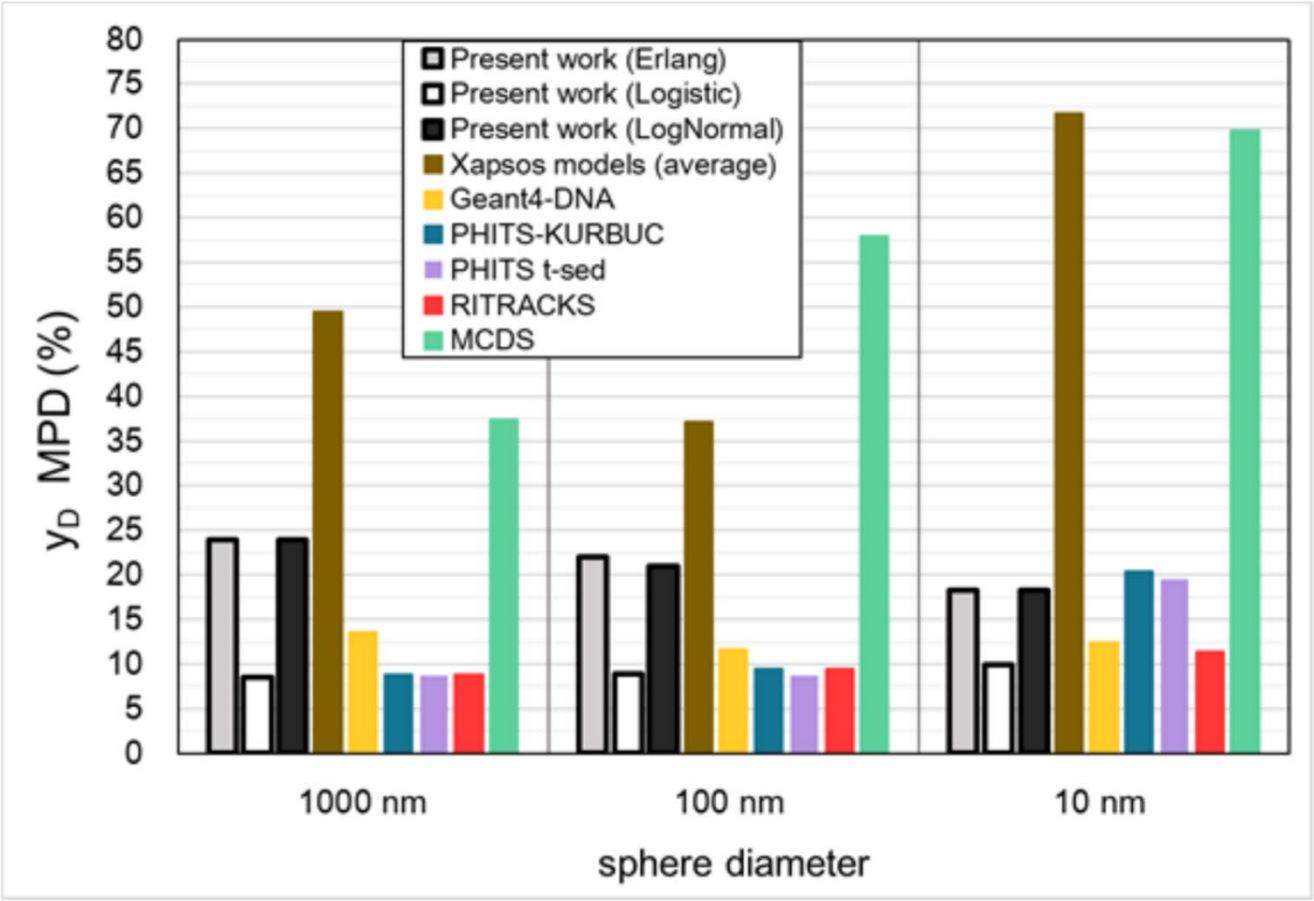
Mean percentage deviation (MPD) calculated by Eq. (34) for each ${y}_{\text{D}}$ dataset. The mean ${y}_{\text{D}}$ of the MCTS codes is used as baseline in the calculation of the MPD

To obtain a single-value indicator of the discrepancy among the various ${y}_{D}$ datasets, the RD is averaged over the proton energies and depicted in Fig. 6 as the mean percentage deviation (MPD):

34 $$MPD = \frac{1}{N}\mathop \sum \limits_{i}^{N} \frac{{|y_{D} \left( {T_{i} } \right) - \overline{{y_{D,MCTS} \left( {T_{i} } \right)}} |}}{{\overline{{y_{D,MCTS} \left( {T_{i} } \right)}} }} \times 100\%$$

where *T*_i_ is the *i*-th discrete energy value in the 1 MeV–1 GeV interval, $\overline{{y }_{\text{D},\text{MCTS}}\left({T}_{\text{i}}\right)}$ is the average ${y}_{\text{D}}$ of the MCTS codes at energy *T*_i_, and *N* is the total number of discrete energies (*T*_i_) considered in this study.

**Fig. 7:**
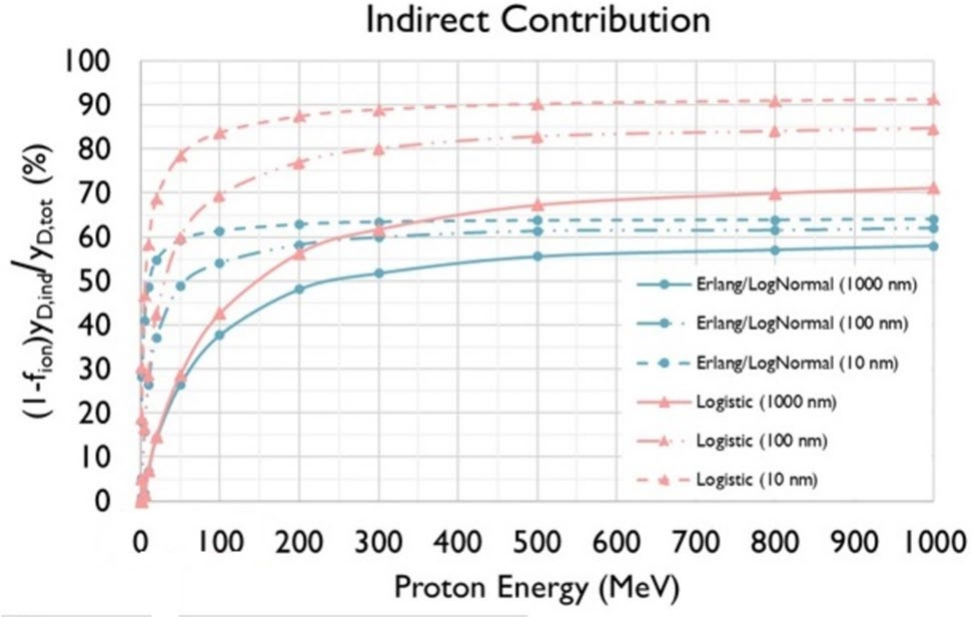
Contribution (%) of the indirect part of the dose-mean lineal energy, ${y}_{\text{D},\text{ind}}$ (see Eq. 6), for the three energy straggling distributions (Erlang, Log-Normal and Logistic) examined in the presented microdosimetric model and the three sphere diameters (1000, 100, and 10 nm). The results for the Erlang and Log-Normal distributions are practically identical

Figure 7 presents the weighted ($1-{f}_{\text{ion}}$) indirect contribution (${y}_{\text{D},\text{ind}}$) to ${y}_{D}$ (see Eq. (6)) relative to the total ${y}_{\text{D}}$ for the present microdosimetric model and the three energy-straggling distributions (Erlang, Log-normal, and Logistic) used in this work, for the three sphere diameters (10, 100, and 1000 nm). Note that the Log-normal and the Erlang distribution yield very similar ${y}_{\text{D}}$ values.

**Fig. 8:**
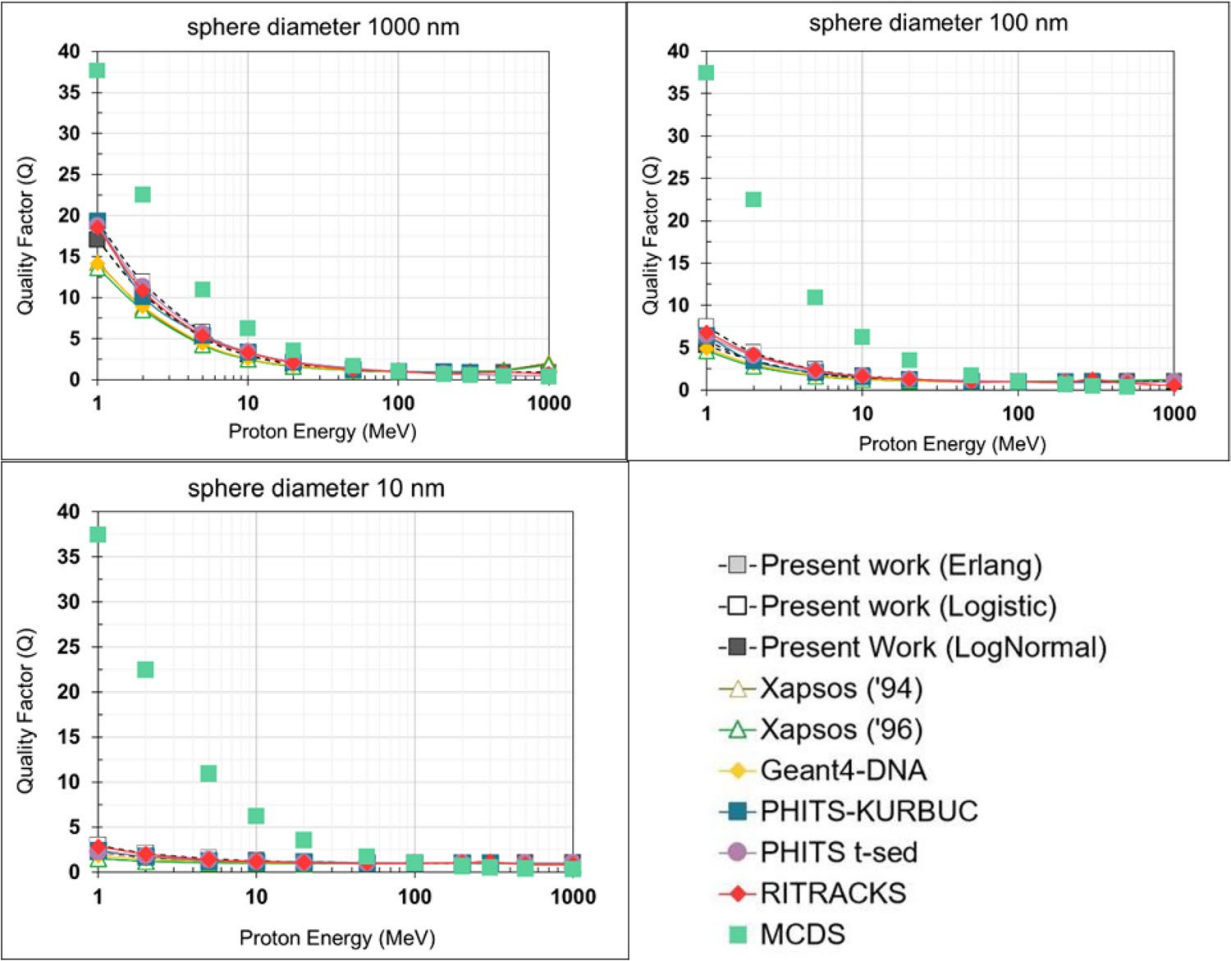
TDRA-based $Q$ values for different liquid water spheres (diameters 10, 100, and 1000 nm) over the proton energy range 1 MeV–1 GeV. The ${y}_{\text{D}}$ data used as input to TDRA are obtained from the analytic models, the MCDS and the MCTS simulations (see Fig. 4). The proton energy at 100 MeV was used for normalization (i.e., $Q$=1 at 100 MeV)

The ${y}_{\text{D}}$ values obtained by the different approaches examined in this work (microdosimetric models, MCDS, and MCTS simulations) were used to calculate the variation of proton Q values over the energy range 1 MeV–1 GeV based on the TDRA approach (Eq. (5)). The TDRA-based Q values are depicted in Fig. 8.

## Discussion

The aim of the present work is the development of an improved microdosimetry-based analytic model for the calculation of proton ${y}_{\text{D}}$ values in sub-micron target volumes (10–1000 nm) over a broad energy range (1 MeV–1 GeV) and the comparison of its performance against MCTS simulations. The dielectric formulation of the RPWBA has been used to determine the model parameters, like the energy-loss straggling factor (${\delta }_{2}$) and the characteristics of the delta-ray spectrum. The RPWBA calculations have been benchmarked against the latest stopping power values for liquid water from ICRU Report 90 (see Fig. 1). The comparison shown in Fig. 1 reveals a good agreement of the present SP calculations with ICRU Report 90, with differences not exceeding 10% over the whole energy range studied (1 MeV–1 GeV).

As it is illustrated in Fig. 2 (left panel) the straggling factor (${\delta }_{2}$) calculated using Eq. (23) is almost independent of proton energy in the case of small spheres (10 nm and 100 nm) but exhibits a sizeable variation with proton energy for the 1000 nm sphere. A previous study(Xapsos et al. 1994) has shown that if the penetration range of the most energetic electrons (${E}_{\text{max}}$) exceeds the target dimensions, then ${\delta }_{2}$ becomes a function of the sphere diameter (or cut-off energy $\Delta$ in this case) but does not depend (or slightly depends) on the ion energy. This is also confirmed in our study (see Fig. 2). Thus, the use of an average ${\delta }_{2}$ value (as in our fitted equation; Fig. 2 right panel) is well justified. However, the straggling factor (δ_2_) has a stronger effect on the energy-loss straggling variance (${V}_{\delta }$) (and on ${y}_{D}$) for high-energy protons (> 10 MeV) and smaller targets (< 100 nm)(Xapsos et al. 1994, 1996). The ${\delta }_{2}$ values calculated in the present work using Eq. (23) are smaller than those obtained by Kellerer’s approach for all sphere diameters. This can be explained by the fact that the present approach is better suited to calculate the small energy transfers (leading to low-energy secondary electrons) as compared to Kellerer’s $1/{E}^{2}$ approximation which only holds for high energy transfers.

The performance of the present analytic model for the calculation of proton ${y}_{D}$ values in sub-micron volumes of liquid water is examined in Figs. 4 and 5 by comparing against the conventional Xapsos models, MCTS simulations (Geant4-DNA, PHITS and RITRACKS), and the MCDS code. At first, we should note that, despite the alternative methodologies (and irradiation geometry-specific factors) used to calculate $y$ as well as the somewhat different interaction models adopted, the MCTS codes are in fair agreement, with differences being overall up to ~10–20% throughout the present energy range (1 MeV–1 GeV) and target dimensions (10–1000 nm) studied. As expected, the largest discrepancies (~20%) among the MCTS codes are observed for the smallest volume of 10 nm, while the agreement improves (~5–10%) as the target volume increases to 100 and 1000 nm. On the other hand, the differences between the MCDS and the MCTS codes are increasing with proton energy (for all target volumes), becoming particularly large (~50–100%) for protons energies above several tens of MeV. These trends are consistent with the fact that the LET-based MCDS dose algorithm neglects energy-loss straggling and delta-ray effects (i.e., delta-ray escape from the target, as well as delta-ray influx to the target from protons passing near the target) which become important with increasing proton energy and decreasing site-size. It is important to note that the simulation of DNA damage in the MCDS does not rely on the embedded dosimetry model; rather, MCDS simulations of DNA damage are based on a probabilistic model calibrated to reproduce the results of MCTS simulations for a range of low and high LET radiations.

The results of Fig. 5 also reveal that, regardless of the energy-loss straggling distribution (Log-Normal, Erlang and Logistic; see Fig. 3), the ${y}_{\text{D}}$ values obtained by the new model are in much better agreement with the MCTS data as compared to the conventional Xapsos models. The latter tend to severely overestimate (by more than 100%) the MCTS data above a few hundred MeV, even for the large target spheres. Importantly, the improved performance of the present model becomes more evident as the sphere diameter decreases, especially for the 10 nm sphere which may be considered biophysically the most relevant. Figure 5 also illustrates that a significant improvement by the present model is caused by the replacement of the Log-Normal by the Logistic distribution to describe the energy-loss straggling. The latter brings the present ${y}_{\text{D}}$ calculations consistently closer to the average ${y}_{\text{D}}$ of the MCTS data, as compared to the use of the Log-Normal (or the Erlang) distributions. The differences between the present model and the Xapsos models can be primarily attributed to the method used to calculate the ${y}_{\text{D}}$ for indirect events (and less so to the ${\delta }_{2}$ values) as can be seen from Fig. 6 under the same energy-loss straggling distribution (i.e., Log-Normal). The strong influence of the indirect contribution (${y}_{\text{D},\text{ind}}$) to the total ${y}_{\text{D}}$ of Eq. (6), is illustrated in Fig. 7.

A simple indicator of the overall performance of the different ${y}_{\text{D}}$ datasets is the MPD value calculated by Eq. (34) and presented in Fig. 6. Note that the arithmetic mean ${y}_{\text{D}}$ value of the MCTS data was used here as the baseline for the MPD calculations. The new model yields a much lower MPD compared to the conventional Xapsos models, irrespective of the straggling distribution used. Specifically, the Xapsos models have an MPD between 37 and 72% whereas the present model has an MPD between 8 and 24%, with the exact value depending upon the straggling distribution used (Erlang, Logistic, or Log-Normal). Specifically, the Logistic distribution yields the lowest MPD (8–10%) whereas the Erlang and Log-Normal distributions yield a somewhat higher MPD (18–24%). Thus, it is recommended that the present model is being used with the Logistic distribution. The deterministic algorithm used in the MCDS has an MPD of 37–70%, comparable to the Xapsos models. Intriguingly, the MPDs of the MCTS codes are in the range of 8–20%, i.e., they are comparable to the MPDs of the present model. In other words, the present analytic model with any of the three straggling distributions, performs (for ${y}_{\text{D}}$ calculations in the examined energy and target-size range) similarly to the examined MCTS codes. It is noteworthy that, especially for the 10 nm sphere which may be most relevant to the simulation of complex forms of DNA damage, the present model with the Logistic distribution exhibits the lowest MPD from all ${y}_{\text{D}}$ datasets studied.

Using the ${y}_{\text{D}}$ datasets presented in Fig. 4, it is straightforward to calculate TDRA-based $Q$ values based on Eq. (5). As seen in Fig. 8, the differences in $Q$ become gradually significant (especially for the MCDS) below a few tens of MeV, for all spherical diameters. At the intermediate energy range (~ 50–500 MeV) the resulting $Q$ values are mostly insensitive to estimates of ${y}_{\text{D}}$ among models. Small (or moderate) discrepancies are also observed at the high energy end (~ 1 GeV). It is noteworthy that the $Q$ values calculated by the present model fall within the range of $Q$ values obtained by the MCTS codes.

## Conclusion

An improved microdosimetry-based analytic model is presented that refines and updates the earlier Xapsos models and allowing for the calculation of the dose-mean lineal energy (${y}_{\text{D}}$) in sub-micron liquid water spheres over a broad range of proton energies (1 MeV–1 GeV) in good overall agreement (within ~ 10%) with state-of-the-art MCTS codes (Geant4-DNA, PHITS, and RITRACKS). Results from the MCDS quasi-deterministic algorithm highlight the limitation of LET-based calculations for sub-micron volumes irradiated by MeV–GeV protons. TDRA-based calculations of the radiation protection quality factor ($Q$) are reported, highlighting its sensitivity to the size of the target volume as well as the ${y}_{\text{D}}$ dataset at low proton energies. It is envisioned that the present model might be used as a reliable alternative to CPU-intensive MCTS simulations for practical calculations of ${y}_{\text{D}}$ (and $Q$) in both medical and space applications with an accuracy comparable to state-of-the-art MCTS codes.

## Data Availability

The datasets generated during and/or analysed during the current study are available from the corresponding author on reasonable request.
